# Idiopathic left ventricular outflow tract ectopy: a single focus with extremely divergent breakouts

**DOI:** 10.1186/1471-2261-14-161

**Published:** 2014-11-18

**Authors:** Sherif Gouda, Dan Wichterle, Petr Peichl, Josef Kautzner

**Affiliations:** Department of Cardiology, Institute for Clinical and Experimental Medicine, Vídeňská 1958/9, Prague, 14021 Czech Republic; Department of Cardiology, Cairo University, Cairo, Egypt

**Keywords:** Ventricular tachycardia, Premature ventricular contractions, Left ventricular outflow tract, Preferential conduction, Non-coronary aortic cusp, Catheter ablation

## Abstract

**Background:**

Idiopathic ventricular tachycardia (VT) and/or premature ventricular contractions (PVCs) arise most commonly from the right ventricular outflow tract and less frequently from the left ventricular outflow tract (LVOT), either below or above the semilunar valves.

**Case presentation:**

We report a case of 24-year-old man with idiopathic ventricular tachycardia from a single focus in the supravalvular left ventricular outflow tract with two extremely divergent breakouts observed during the ablation procedure.

**Conclusion:**

Focal sources of ventricular arrhythmia in the aortic root may have different preferential exits and meticulous activation sequence mapping is the preferable strategy to delineate the site of origin.

## Background

Idiopathic ventricular tachycardia (VT) and/or premature ventricular contractions (PVCs) arise most commonly from the right ventricular outflow tract and less frequently from the left ventricular outflow tract (LVOT), either below or above the semilunar valves. Therefore, some arrhythmias from the LVOT can be targeted via aortic sinuses of Valsalva - more often from the left coronary cusp (LCC) than the right coronary cusp (RCC), and very rarely from the non-coronary cusp (NCC) [1–4]. We report a case with idiopathic VT/PVCs from a single focus in the supravalvular LVOT with two extremely divergent breakouts observed during the ablation procedure.

## Case presentation

**Figure 1:**
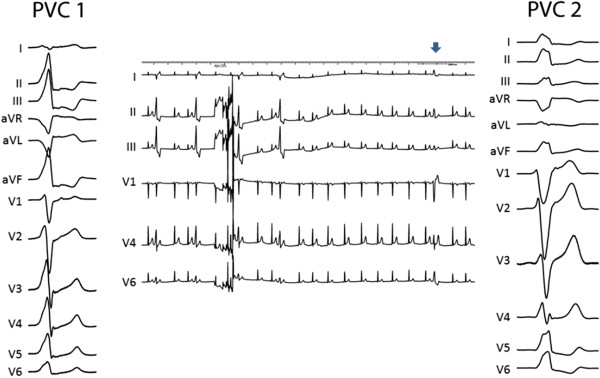
**The left and right panels show the 12-lead ECG of the PVC-1 and PVC-2 before and after initial ablation at the LCC, respectively.** The middle panel shows disappearance of PVC-1 within few seconds after the beginning of ablation with subsequent appearance of PVC-2 eleven seconds later (blue arrow). Note the change in R wave amplitude in inferior leads and the shift of transition zone from V2/3 to V4.

A 24-year-old man, working as a professional dancer, was referred for investigation of recurrent syncopal episodes that were related to physical exercise. His 12-lead surface ECG revealed frequent monomorphic PVCs suggestive of LVOT origin (early precordial transition before V3, high R wave amplitudes in inferior leads, QS in aVL more negative than in aVR, and deep S in lead I) (PVC-1 in Figure 1). According to Holter monitoring, PVCs accounted for 20% of all QRS complexes and sporadic episodes of non-sustained VT of the same morphology were also documented. Physical examination was unremarkable and transthoracic echocardiography and cardiac magnetic resonance imaging studies excluded structural heart disease.

**Figure 2:**
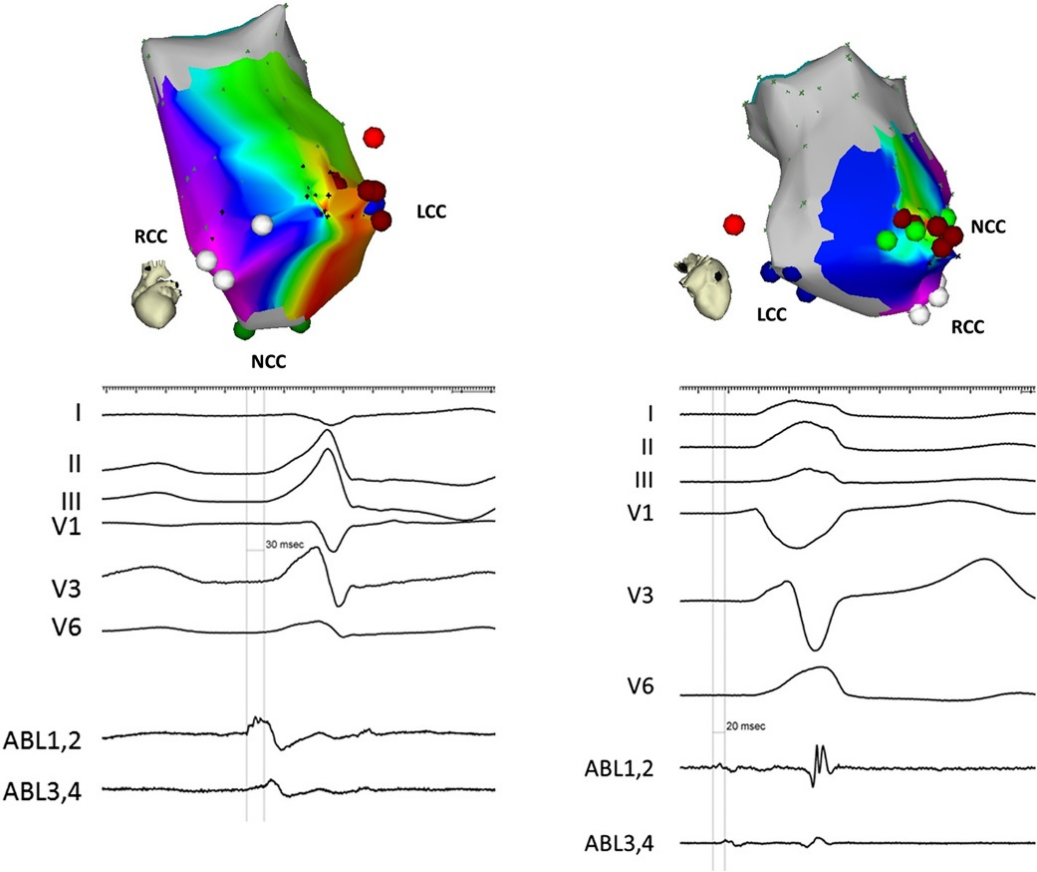
**The left panel shows electroanatomic activation map of the aortic root for PVC-1 with the earliest activation and ablation site at the LCC (left anterior oblique view).** Corresponding local bipolar electrogram (prematurity of 30 ms) is shown below. The right panel shows posterior view of aortic root map for PVC-2 with the earliest activation and ablation site at the NCC. Corresponding local bipolar electrogram (prematurity of 20 ms) is shown below. The bright red CARTO point denotes ostium of the left main coronary artery. Abbreviations: ABL 1.2 and ABL 3.4 – distal and proximal bipoles of the ablation catheter, LCC/RCC/NCC – left/right/non coronary cusp.

An electrophysiological study and ablation procedure was performed aiming at ablation of the focal source. At baseline, the monomorphic PVCs were frequent. Activation mapping and pace mapping in the LVOT was performed using ablation catheter (Navi-Star™ ThermoCool™, Biosense Webster Inc.) introduced retrogradely via the right femoral artery. A three-dimensional shell of the aortic root was created using CARTO system (CARTO XP™, Biosense Webster, Diamond Bar, CA, USA). Tagging of mid-portions of individual aortic sinuses and the ostium of the left main coronary artery (LMCA) was performed with the guidance of intracardiac echocardiography (ICE) (AcuNav™ ultrasound catheter, Sequoia echocardiograph, Siemens Acuson, Mountain View, CA, USA). The earliest ventricular electrograms were obtained in the middle portion of the left coronary cusp (LCC) with prematurity of 30 ms before the QRS onset (Figure 2) and excellent pace map. This site was about 15 mm away from the ostium of LMCA. Radiofrequency ablation (power-control mode at 25 W with an irrigation flow rate of 20 mL/min) was performed at this site during ventricular trigeminy. Ectopy was abolished within 4–5 seconds of radiofrequency current delivery. However, 11 seconds later, PVCs of different, so far not observed, morphology (PVC-2) appeared with left bundle branch block pattern, precordial transition between V3 and V4, much lower R wave amplitude in inferior leads, monophasic R in lead I and slightly positive QRS in lead aVL (Figure 1).

**Figure 3:**
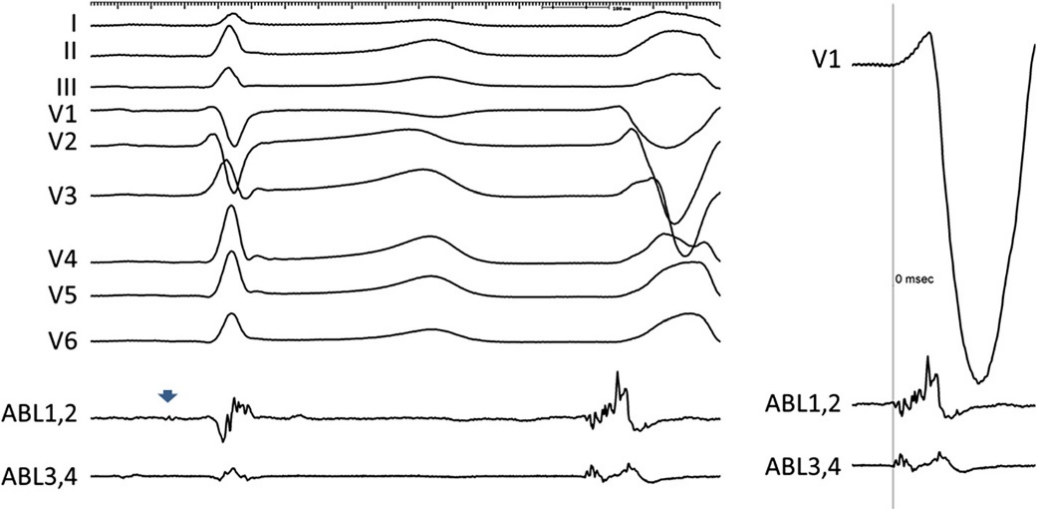
**The left panel shows the signal from ablation catheter at the His position during activation mapping of PVC-2.** Note the His potential at the distal bipole during sinus beat (blue arrow). The right panel shows in magnification the timing of the corresponding local electrogram during PVC-2 indicating zero prematurity in relation to the QRS onset and absence of activation gradient between distal and proximal bipoles. Abbreviations as in Figure 2.

Initially, PVC-2 was noticeably less frequent than the original ectopy (PVC-1) but still amenable for activation sequence mapping. The earliest signal was detected in the right ventricle at the His region with excellent pace map but zero prematurity in relation to the QRS onset and absent activation gradient between proximal and distal bipoles of the mapping catheter (Figure 3). When re-mapping was commenced in the aortic root, local activation during ectopy was considerably late in both LCC and RCC. Thorough mapping of NCC showed low amplitude, but the earliest, ventricular signal in the middle portion of the cusp with prematurity of 20 ms before the QRS onset (Figure 2). Radiofrequency current application at this site, using the same power and irrigation settings as described above, immediately abolished the ectopy which did not recur during the waiting time of 20 minutes including an isoproterenol challenge. At the 4-month follow-up visit, the patient was asymptomatic and 24-hour Holter monitoring revealed only 40 monomorphic PVCs with reversed axis compared to sinus QRS complexes, unlikely to originate from LVOT.

Aortic root anatomy and electrophysiological properties of surrounding myocardium may explain the above observation. In addition to the crescents of ventricular musculature at the base of sinuses of great arteries, significant number of patients may have ventricular myocardial extensions beyond the ventricular–arterial junction and even beyond the semilunar valves. These extensions may course in either an oblique or longitudinal manner, may be continuous or discontinuous with the underlying ventricular musculature and may be located within the adventitia or on its outer side (epicardial surface). Myocyte hypertrophy and fibrosis, as well as interposed adipose tissue are commonly found with ventricular myocardial extensions [5]. Because of the complex nature of these myocardial extensions, focal origin of arrhythmia may have variable exits across the circumference of the semilunar valve.

Several reports described the concept of preferential conduction between site of origin of outflow tract arrhythmia and outbreak site into larger mass of myocardium [6–9]. In addition, few cases of successful ablation of the arrhythmogenic focus within the NCC have been reported [10–13]. To the best of our knowledge this case is the first description of two opposite exits into ventricular myocardium from the region of the commissure between the LCC and NCC. The most plausible explanation is the presence of a single focus within the myocardial extensions into the LCC/NCC commissure with one exit ablated within the LCC, unmasking conduction in the opposite direction via the NCC to the right ventricular myocardium at the His bundle region.

Another possible explanation would be the existence of two different arrhythmogenic foci in both LCC and NCC. This hypothesis might be supported by different coupling intervals - 510 and 620 ms for PVC-1 and PVC-2, respectively. In such a case, the clinical appearance of the second focus could have been fully suppressed by the earlier activity from the first focus until this one was eliminated by ablation. However, the presence of two closely related foci with similar ectopic activity and different coupling intervals appears to be less likely than our hypothesis on a change of the exit. In fact, the different coupling intervals of PVC-1 and PVC-2 from the same focus can be easily explained. Provided that there is a slowly conducting zone between both ablation sites at LCC and NCC and, more importantly, that the focus is located asymmetrically in this zone (distinctly closer to the LCC ablation site), then the change in exit route is essentially associated with prolongation of coupling interval of PVC. Those who are mathematics fans can surely estimate that the conduction time from the focus to NCC versus LCC ablation site should have been longer by ~65 ms to explain the coupling interval change of ~110 ms taking into account comparable reversal of signals during SR at both ablation sites.

The phenomenon of preferential conduction, which played a substantial role in our case, speaks of the complexity of ventricular myocardial extensions both at the structural level (extent of these extensions and their connections) and functional level (conduction properties and refractoriness). Despite the general absence of ventricular musculature at the base of NCC due to the aorto-mitral continuity, the case demonstrates the slowly conducting interconnection between myocardial networks adjoining both LCC and NCC. It is also likely (although we have no direct evidence) that the focus was not ablated directly but merely isolated by creating conduction block at the only two potential exit routes. In general, the property of preferential conduction may cause PVCs from the LVOT to show variable electrocardiographic features according to the breakout sites. This adds to the limitations of ECG algorithms and pace mapping to predict the site of origin of LVOT arrhythmias.

Concerning the change between PVC-1 and PVC-2 morphology, the decrease in R wave amplitude in the inferior leads was the most prominent, therefore such a change during radiofrequency ablation for outflow tract arrhythmia should prompt further mapping to search for another target in a different portion of the outflow tract [6]. In contrast to our case, less prominent change in QRS morphology after ablation of LVOT arrhythmias was previously reported. This can be explained by multiple potential breakouts in other portions of the LVOT, for example in the region of LCC/RCC commissure, where ventricular myocardial extensions are more abundant (and all are connected to the LV myocardium) compared to the myocardial network at LCC/NCC region. In the previously reported cases of successful ablation of ventricular arrhythmia in the NCC, significant variations in the QRS morphology with variable precordial transition and inconsistent QRS morphology in aVL ranging between QS pattern, slightly positive QRS and monophasic R were observed. Although anatomical differences of individual patients and varying ECG electrode positioning may play a role, contributions from more than one exit site to QRS morphology could, at least partly, explain these variations depending on the volume of myocardium activated by each breakout site. For example, if the posterior exit site toward the His region is dominant, the QRS morphology will resemble right-ventricular arrhythmias arising from the proximity of membranous septum, on the other hand, if the anterolateral exit site to the subvalvular LVOT is dominant, the QRS will indicate more typical LVOT morphology, and if both exits participate equally, the intermediate fusion QRS morphology would be expected.

In our case, single catheter approach was utilized to map different areas of interest sequentially with the help of the electroanatomic mapping system and ICE guidance. The use of ICE allowed real-time visualization of the exact catheter position within the aortic cusps and their commissures. It also enabled us to identify the distance of ablation catheter tip from the coronary ostia in order to prevent complications. This case highlights the importance of mapping the NCC and RCC when RV mapping reveals the earliest activation at the His bundle region, as this will reduce risk of inadvertent damage to the AV conduction system.

## Conclusions

In conclusion, this case demonstrates that focal sources in the aortic root may have different preferential exits and that meticulous activation sequence mapping is the preferable strategy to delineate the site of origin. ICE appears to be helpful in precise positioning of the mapping catheter within the coronary cusps and commissures. Preferential conduction may cause PVCs from the LVOT to show variable ECG features and limit the use of ECG algorithms and pace mapping to predict the site of origin. Rare cases of successful ablation of ventricular arrhythmia at the NCC might not indicate true rarity of the NCC involvement because tiny ventricular electrogram at the NCC can easily be missed.

## Consent

Written informed consent was obtained from the patient for publication of this Case report and any accompanying images. A copy of the written consent is available for review by the Editor of this journal.

## Abbreviations

ICE: Intracardiac echocardiography
LCC/RCC/NCC: Left/right/non coronary cusp
LMCA: Left main coronary artery
LVOT: Left ventricular outflow tract
PVC: Premature ventricular contraction
VT: Ventricular tachycardia.
